# Extraction Optimization, Antioxidant Capacity and Phenolic Profiling of Extracts from Flesh, Peel and Whole Fruit of New Zealand Grown Feijoa Cultivars

**DOI:** 10.3390/antiox8050141

**Published:** 2019-05-21

**Authors:** Yaoyao Peng, Karen Suzanne Bishop, Siew Young Quek

**Affiliations:** 1 Food Science, School of Chemical Sciences, The University of Auckland, Auckland 1010, New Zealand; ypen083@aucklanduni.ac.nz; 2 Discipline of Nutrition and Dietetics, School of Medical Science, Faculty of Medicine and Health Science, The University of Auckland, Auckland 1023, New Zealand; k.bishop@auckland.ac.nz; 3 Riddet Institute, New Zealand Centre of Research Excellence for Food Research, Palmerston North 4474, New Zealand

**Keywords:** feijoa extracts, extraction optimization, orthogonal design, cultivar difference, antioxidant activity, phenolic compounds, LC-ESI-MS/MS

## Abstract

Feijoa fruit is becoming increasingly popular, yet limited studies have focused on the antioxidant capacity and phenolic profiling of its extracts. In this research, optimization of phenolic extraction from feijoa flesh, peel, and whole fruit from four New Zealand grown cultivars was conducted using orthogonal design. Antioxidant activities of the extracts were assessed, followed by phenolic profiling by a validated liquid chromatography-electrospray ionization-tandem mass spectrometry (LC-ESI-MS/MS) method. For feijoa flesh and whole fruit, the extraction was optimized using 70% ethanol, material to solvent ratio of 1:30, at extraction temperature of 50 °C for 30 min. For feijoa peel, extraction at 50 °C for 60 min using 50% ethanol with a material to solvent ratio of 1:30 were the optimized conditions. Results showed feijoa peel had higher total phenolic content (TPC) and antioxidant activities than the flesh and whole fruit. Overall, the Unique cultivar had a relatively higher TPC and antioxidant activity than the other cultivars tested. A total of 15 phenolic compounds were identified, and seven of them were reported for the first time in feijoa fruit. This is the first systematic investigation on the extraction method, phenolic content, antioxidant activity and phenolic profile of feijoa emphasis on comparison of sample types and cultivars.

## 1. Introduction

The exploration of health benefits of fruits and vegetables continues unabated. One of the major bioactivities of the extracts from fruits and vegetables is antioxidant activity, and studies have revealed that plant-derived phenolic compounds, including phenolic acids, flavonoids, and tannins, are an important group of natural antioxidants [1,2]. Due to the well elucidated association between the enhanced oxidative stress and numerous diseases, such as cardiovascular and metabolic diseases [3], Alzheimer disease [4] and cancers [5], as well as aging [6], antioxidants are essential in disease prevention and treatment, as well as health promotion and anti-aging. 

Feijoa (*Acca sellowiana* (O. Berg) Burret) is a well-known fruit in New Zealand and is being rapidly introduced to countries worldwide. The plant is native to South America, much of which provides a special “cool winter and dry summer” climate for it to produce good fruits [7]. The feijoa fruit has a green skin and a jelly pulp and is famous for its unique aroma and flavor. Within the last few decades, increasing attention has been paid to the investigation of its volatile compounds [8,9,10], health benefits [11,12,13,14,15] and bioactive compounds [16,17,18,19,20,21]. It is believed that feijoa fruit harbor significant antioxidant activities [22], and the phenolic compounds could be the major contributors to its bioactivities [23]. However, despite these insights, there is still much to be added to the “feijoa database” regarding its bioactivity and bioactive compound identification. 

Literature has shown that feijoa fruits of different cultivars may differ in their physiochemical, nutritional, sensory, and bioactive properties [11,23,24]. In addition, similar to other fruits [25,26,27], the peel, flesh and whole fruit from feijoa fruit could also vary significantly regarding the bioactivities and constitution of bioactive compounds. However, no existing study has systematically compared the antioxidant activity and phenolic profile of feijoa extracts from the flesh, peel and whole fruit, from different cultivars.

Many factors can affect the extraction efficiency of phenolic compounds from plant materials. Commonly assessed factors include solvent type, extraction time, sample to solvent ratio, and extraction temperature [28,29]. Proper optimization of the extraction conditions can greatly improve extraction efficiency. Traditional optimization using a single factor experiment to determine the optimal level of each extraction factor under set conditions is not adequate to distinguish cross-effects of different factors. The orthogonal design has the advantage to combine different levels of each factor randomly, and the optimal level, influential order and the significance of the factors can be determined by statistical analysis. The method has been successfully applied in the extraction optimizations of phenolic compounds [29,30].

Qualification and quantification of phenolic compounds in plant extracts are essential in view of their potential applications in functional food and pharmaceutical areas. The liquid chromatography (LC) method is one of the most frequently used techniques for compound characterization, and the LC coupled with mass spectrometry (MS) further enhances the accuracy and sensitivity of compound detection and identification. Moreover, the combination of LC-electrospray ionization (ESI)-MS/MS has been widely applied in rapid screening of complex sample matrixes [31,32,33], due to the highly specific ion transition mode of the method that is capable of detecting the compound of interest with high accuracy. 

The objective of this study was to optimize the extraction method for phenolic compounds from feijoa flesh, peel and whole fruit, and to assess the total phenolic content and antioxidant capacity, as well as to establish a phenolic profile of feijoa extracts. Even though two previous studies [19,21] have conducted the phenolic extraction from feijoa and tested the TPC and antioxidant activities, however, the flesh, peel and whole fruit of feijoa were systematically compared in neither of these studies nor were the different feijoa cultivars compared. Thus, our study was the first to report the differences across feijoa sample types and cultivars with regards to phenolic content and antioxidant activity. 

## 2. Materials and Methods 

### 2.1. Sample Preparation

Fresh feijoa fruits of Apollo, Unique, Opal Star and Wiki Tu cultivars, at the commercial ripening stage, were kindly provided by local orchards in the Northern island of New Zealand. Feijoas were washed carefully to remove external attachments upon arrival at the Food Science laboratories at the University of Auckland. Feijoa flesh and peel were separated using a manual peeler, and the flesh and whole fruit samples were cut into 1 cm-thick slices. The prepared feijoa samples were kept at −20 °C for 12 h followed by storage at −80 °C for 4 h, and then transferred to a freeze drier (Labconco, USA). After 72 h of freeze drying, the freeze-dried feijoa slices were further ground into a fine powder using a coffee grinder (Breville BCG200, Sydney, Australia) and the obtained samples were stored at −80 °C until required. 

### 2.2. Chemicals

Gallic acid, caffeic acid, ellagic acid, quercetin, flavone, catechin, epicatechin, epigallocatechin, epicatechin gallate, epigallocatechin gallate, (±)-6-hydroxy-2,5,7,8-tetramethylchromane-2-carboxylic acid (Trolox), 2,2′-diphenyl-1-picrylhydrazyl (DPPH), 2,4,6-tris(2-pyridyl)-s-triazine (TPTZ), and the Folin-Ciocalteu reagent were purchased from Sigma-Aldrich (St. Louis, MO, USA). Quercetin-3-galactoside, quercetin-3-rhamnoside, myricetin-3-*O*-galactoside, myricetin-3-*O*-glucoside, procyanidin B1 and procyanidin B2 were supplied by Extrasynthese (Genay, France). All chemicals and reagents were of analytical grade unless specified. 

### 2.3. Orthogonal Design 

Four extraction factors namely extraction temperature (A), ethanol concentration (B), extraction time (C), and material to solvent ratio (D) were optimized using an L9 (3^4^) orthogonal design (Table 1), according to Wu, et al. [28] with modifications. Three levels (level 1, 2, 3) of each extraction factor were selected based on a preliminary screening using single factor experiments. 

### 2.4. Total Phenolic Content Assay

Total phenolic content (TPC) was employed as one of the response factors in the orthogonal design to evaluate the extract efficiency of phenolic compounds from feijoa samples. TPC was conducted by the Folin-Ciocalteu (FC) Assay using the 96 well plates according to Medina-Remón, et al. [34]. A linear standard curve (R^2^ > 0.99) was plotted using a serial concentration (0.2 mg/mL, 0.1 mg/mL, 0.08 mg/mL, 0.06 mg/mL, 0.04 mg/mL 0.02 mg/mL and 0.01 mg/mL) of gallic acid. The TPC of each sample was calculated according to the standard curve and expressed as milligrams of gallic acid equivalents per gram dry weight of feijoa samples (mg GAE/g dw).

### 2.5. Antioxidant Assays

Two antioxidant assays, namely DPPH and ferric reducing antioxidant power (FRAP), were utilized as response factors in the orthogonal design, as well as to assess the antioxidant capacity of feijoa extracts. The DPPH and FRAP assays were carried out according to the methods of Herald, et al. [35] and Benzie and Strain [36], respectively. Serial concentrations (50 μM, 100 μM, 150 μM, 200 μM, 400 μM, 600 μM and 800 μM) of trolox standard were prepared to establish a linear standard curve (R^2^ > 0.99). The antioxidant activity of feijoa extract was calculated according to the standard curve and expressed as micromoles of Trolox equivalents per gram of dry weight of feijoa samples (μM TE/g dw).

### 2.6. LC-ESI-MS/MS Qualification and Quantification

The phenolic compounds in feijoa crude extracts were qualified and quantified using LC-ESI-MS/MS. An Agilent 1290 Infinity liquid chromatography (LC) system with diode array detector (DAD) (Agilent Technologies, Wilmington, DE, USA), coupled with the Agilent 6460 triple quadrupole mass spectrometer (MS) (Agilent Technologies, Wilmington, DE, USA) with electrospray ionization (ESI) source was employed for this purpose.

The LC was performed using a Reversed-phase Poroshell 120 EC-C18 column (2.1 mm × 100 mm; 4 μm; Agilent Technologies, Wilmington, DE, USA) with mobile phase A and B being 0.2% formic acid in water and 0.2% formic acid in acetonitrile, respectively. The gradient program was optimized as follows: 0 min, 5% B; 2 min, 6% B; 12 min, 7% B; 16 min, 9% B; 26 min, 10% B; 27 min, 11% B; 28 min, 15% B; 30 min, 20% B; 40 min, 60% B; 45 min, 5% B. The injection volume was 3 μL, the column temperature was 35 °C, and the flow rate was 0.5 mL/min. 

The MS collision energy was adjusted for each standard compound to produce daughter ions by ESI source using a multiple reaction monitoring (MRM) mode. The optimized MS program conditions were 3.5 kV of capillary voltage, nebulizer gas at 45 psi, drying gas at 250 °C and 10 L/min of flow, and sheath gas with a temperature at 280 °C and flow at 11 L/min.

Quantifications of phenolic compounds in feijoa extracts were carried out using standard calibration curves. Each standard curve of the reference standards was established according to the correlation between the peak area (1/10,000) of the selected parent or daughter ion (*y*-axis) and the concentration of the standard compound (*x*-axis). 

Method validation was conducted by evaluating the correlation (R^2^) of the linearity, limit of detection (LOD), limit of quantification (LOD), inter-day and intra-day precision, and recovery, according to Jiao, et al. [37].

### 2.7. Statistical Analysis

All tests were conducted in triplicate. Statistical significance (*p* < 0.05) was determined by one-way analysis of variance (ANOVA) using SPSS 22.0 (IBM Corp., Armonk, NY, USA).

## 3. Results and Discussion

### 3.1. Optimization of Extraction Conditions by Orthogonal Design

Four factors each with three levels were included in the extraction optimization of phenolic compounds from feijoa flesh, peel and whole fruit, using an L9 (3^4^) orthogonal design. As shown in Table 1, nine experimental groups were carried out and evaluated by three response factors including TPC and antioxidant activities. 

The orthogonal design has been successfully applied in extraction optimizations of aimed compounds from various materials [28,29,30,38]. This method has the advantage of requiring fewer experiments than a comprehensive experimental design. In the case of four-factors with a three-level design in the current study, only nine groups of experiments, other than the comprehensive 64 groups, was needed. It has, therefore, greatly shortened the experimental process, as well as decreased solvent and energy consumption. 

The optimal extraction conditions were determined according to the range analysis and ANOVA analysis of the results obtained from the nine orthogonal experimental groups (Table 2). From the range analysis, the optimized extraction conditions could be determined using the combination of the best level for each factor. However, to ensure that the selected level from the range analysis was significantly better than the other levels, ANOVA analysis of each factor was performed to obtain an *F* value [28]. For example, the optimized extraction conditions to obtain the maximum TPC of feijoa whole fruit by the range analysis was A3B3C1D1 (Table 2). However, only factor B (ethanol concentration) and C (extraction time) were shown to be statistically significant (*p* < 0.05) from the ANOVA analysis (Table 2). Thus, the optimal conditions for the maximum TPC of feijoa whole fruit should be adjusted to A1B3C1D1 (extraction temperature of 50 °C, extraction solvent of 70% ethanol, extraction time of 30 min and material to solvent ratio of 1:30), since temperature (factor A) effect could be ignored.

In addition, the optimal extraction conditions, after range analysis and ANOVA analysis, were generally in agreement except for feijoa peel. A combination of A1B3C1D1 was determined to achieve the maximum TPC and antioxidant activities for both feijoa flesh and whole fruit extracts, while combinations of A1B2C2D1 and A1B2C1D1 were optimized for a maximum TPC and the highest antioxidant activities, respectively (Table 2). Similar findings of slightly varied conditions for different optimization purposes were reported by Zhou, et al. [38] working on the extraction optimization of antioxidant peptides from corn using orthogonal design. The final optimal conditions, based on a balanced evaluation of all factor levels, were determined as A1B2C2D1 for feijoa peel. In summary, the optimized extraction conditions for flesh and whole fruit were: extraction temperature of 50 °C, extraction solvent of 70% ethanol, extraction time of 30 min and material to solvent ratio of 1:30; and the extraction at 50 °C using 50% ethanol for 60 min with a material to solvent ratio of 1:30 were the optimal conditions for feijoa peel.

Ethanol concentration was the most significant factor influencing the extraction efficiency of the phenolics and antioxidants from feijoa flesh, peel and whole fruit (Table 2). The extraction solvent is an important factor in the optimization of extraction efficiency. A number of solvents including methanol, ethanol, and acetone, were employed in the extraction of phenolics and antioxidants from plants [39,40,41]. However, compared to methanol and acetone, an aqueous mixture of ethanol is widely accepted in the food industry due to its low toxicity and cost [28]. Therefore, our study has employed ethanol as the extraction solvent and has determined the optimal ethanol concentrations as 70% for feijoa flesh and whole fruit, and 50% for feijoa peel. The polarity of the aqueous ethanol solvent decreased with the increasing concentration of ethanol in the mixed aqueous system. A higher ethanol concentration required in the phenolic extraction from feijoa flesh and whole fruit (consisting predominantly of flesh) suggested that the phenolic compounds in feijoa flesh have higher polarity than those found in feijoa peel. 

Extraction time is another important factor that significantly affected the extraction efficiency of phenolics and antioxidants, particularly in feijoa peel and whole fruit (*p* < 0.05, Table 2). It is noted that the optimal extraction time for phenolics (TPC, *p* < 0.05) and antioxidants (DPPH) from feijoa peel was 60 min required by the range analysis, however, only 30 min of extraction was needed to achieve optimum extraction of phenolics and antioxidants from feijoa flesh and whole fruit. This could be explained by the composition and structure of different feijoa samples. As peel contains more fiber and lignin than flesh, longer extraction time was required to break down the plant cell wall.

Prior to this study, Tuncel and Yılmaz [19] optimized the extraction of phenolic compounds from feijoa flesh. Their optimized conditions were: 80% acetone, material to solvent ratio of 1:60, extraction temperature of 40 °C and extraction time of 3 h. These extraction conditions were further applied to phenolic extraction from peel samples without further optimization. Our results suggest that the optimized extraction conditions for flesh and peel could vary significantly. A lower material to solvent ratio and shorter extraction time than those identified by Tuncel and Yilmaz were also presented in the current study, which makes the extraction procedure more energy saving and environmentally friendly. Furthermore, the utilization of ethanol in this study, as mentioned above, is a safer choice for food application than acetone. 

### 3.2. Total Phenolic Content and Antioxidant Activity of Feijoa Extracts from Four Cultivars

**Figure 1:**
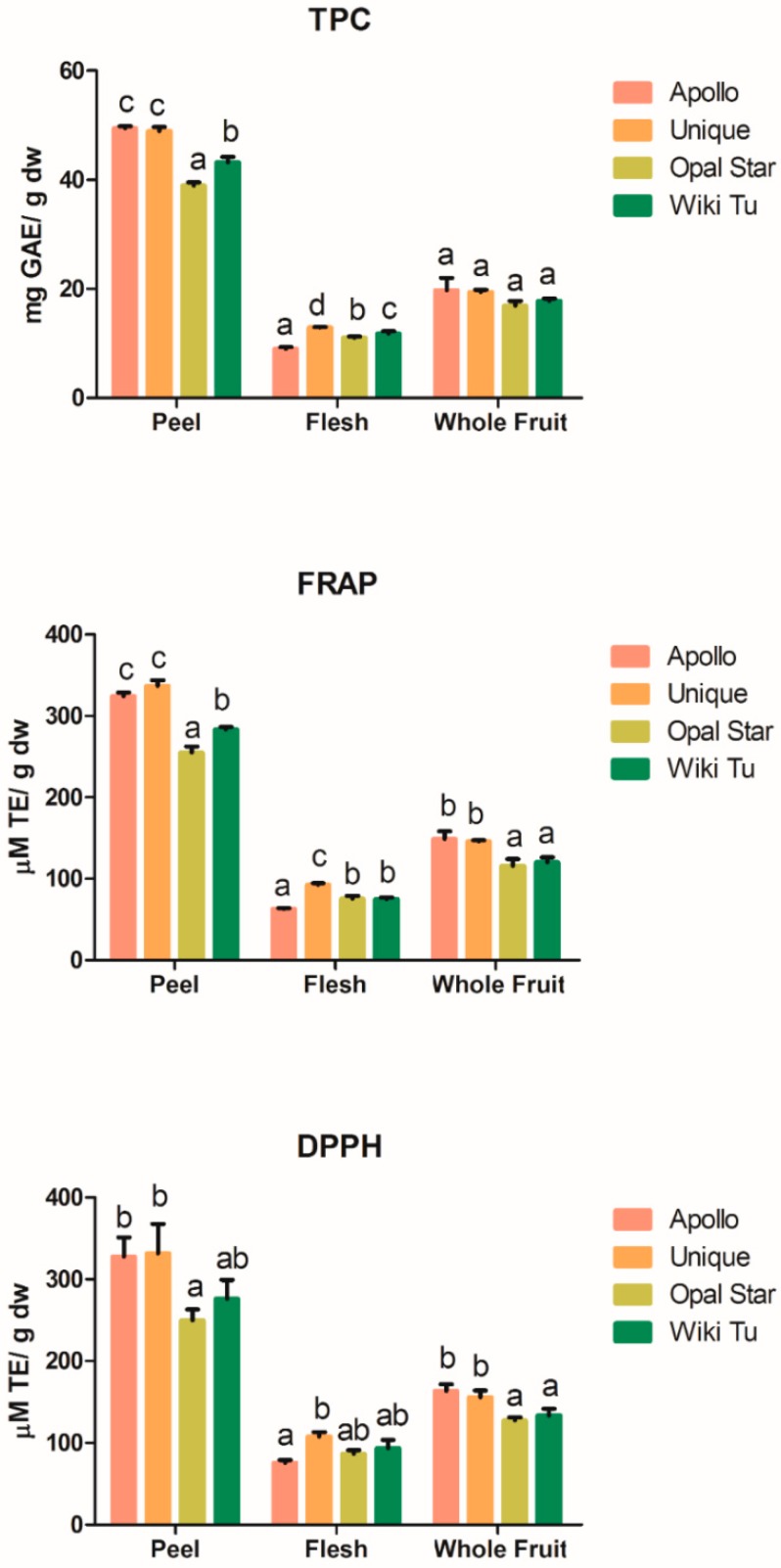
Total phenolic content and antioxidant activity of feijoa extracts from flesh, peel and whole fruit from four cultivars (Apollo, Unique, Opal Star and Wiki Tu). TPC, total phenolic content; GAE, gallic acid equivalent; TE, Trolox equivalent; dw, dry weight; FRAP, ferric reducing antioxidant power; DPPH, the 2,2′-diphenyl-1-picrylhydrazyl assay; abcd, indicators for significance among cultivars (*p* < 0.05).

The optimized conditions from the orthogonal design were applied in the extraction of phenolic compounds from the flesh, peel and whole fruit from four New Zealand grown feijoa cultivars (Apollo, Unique, Opal Star and Wiki Tu). The TPC and antioxidant activities of the extracts were tested and compared among cultivars. Results (Figure 1) show peel extracts had significantly (*p* < 0.05) higher TPC and antioxidant activity than the flesh and whole fruit extracts, while flesh extracts possessed the lowest TPC and antioxidant capacity among the three sample types. Similar findings were observed in a previous study on the antioxidant activity of apple peel and flesh from 11 cultivars [27]. Another study on mango also reported that the peel extracts exhibited stronger antioxidant and anti-proliferative activity than the flesh extracts [42]. The skin of the fruit was a natural protection to the inner nutritious flesh against insects [43], and phenolic compounds have been found to possess significant antimicrobial properties [44,45]. In addition, feijoa extracts have been reported to possess antibacterial and antifungal activities [12,14,46], and our earlier study also demonstrated that feijoa peel extracts induced higher cytotoxicity than the flesh and whole fruit extracts on the selected cell lines [11]. This indicated that the observed high phenolic content in feijoa peel could be responsible for the potential antimicrobial activities, as well as the cytotoxicity. 

Significant differences in the TPC and antioxidant activities were observed among the four tested cultivars. As shown in Figure 1, the TPC and antioxidant activity (from FRAP assay) of the peel extracts from the Apollo and Unique cultivar were both significantly (*p* < 0.05) higher than those of the Opal Star and Wiki Tu peels. Similar phenomena were observed from the whole fruit extracts, where the Apollo and Unique cultivars also had higher TPC and significantly (*p* < 0.05) stronger antioxidant activities than those of the Opal Star and Wiki Tu cultivars. Regarding the flesh extracts, the Unique cultivar was found to have the highest TPC (*p* < 0.05) and antioxidant activities by FRAP assay (*p* < 0.05), followed by the Wiki Tu, Opal Star, and Apollo cultivars (in descending order) (Figure 1). Among the four tested cultivars, it is noted that the Unique cultivar could be the most bioactive, as its peel, flesh and whole fruit extracts possessed relatively high TPC (48.90 ± 0.80, 12.87 ± 0.11 and 19.41 ± 0.43 mg GAE/g dw, respectively) and antioxidant activities (337.01 ± 6.66, 92.45 ± 2.16 and 145.76 ± 1.67 μM TE/g dw, respectively, from FRAP assay). The extracts from Apollo peel and whole fruit had a relatively high TPC and antioxidant activities similar to those of the Unique cultivar, however, the Apollo flesh extracts turned out to contain the lowest TPC (*p* < 0.05) and antioxidant activity (*p* < 0.05 from FRAP assay) among the four cultivars. Thus, the phenolic compound composition of flesh and peel varies among feijoa cultivars, and the TPC and antioxidant activity of feijoa whole fruit extracts were largely contributed by the peel.

The TPC and antioxidant capacity observed in feijoa extracts (Figure 1) were comparable to, if not higher than, what has been determined in other fruit extracts. The TPC of the extracts of strawberry, raspberry, gooseberry, cranberry, cowberry, cloudberry, and bilberry reported by Kähkönen, et al. [47] were 16–24 mg GAE/g dw, 27–30 mg GAE/g dw, 13.2 mg GAE/g dw, 22 mg GAE/g dw, 26–28 mg GAE/g dw, 15–18 mg GAE/g dw, and 33–38 mg GAE/g dw, respectively. The antioxidant activity (from the FRAP assay) of different kiwifruit cultivars tested by Park, et al. [48] ranged from 11 to 94 μM TE/g dw. The averaged TPC and antioxidant activity (FRAP assay) of tomato extracts, from 20 cultivars and breeding lines, were 6.46 mg GAE/g dw and 280.07 μM TE/g dw [49]. The superior phenolic content and antioxidant activity of feijoa suggest the fruit has great potential to be developed into functional foods. In addition, the TPC and antioxidant activities observed in feijoa peel, flesh and whole fruit extracts were positively correlated, which is consistent with numerous studies [50,51,52,53]. This indicates that the phenolics in feijoa were the major contributors to its antioxidant activities. 

### 3.3. Phenolic Profile of Feijoa Extracts from Four Cultivars

#### 3.3.1. Method Validation of LC-ESI-MS/MS Under MRM Mode

Crude extracts from plants normally contain numerous compounds, and in many cases, compound separation by HPLC-DAD may be poor. Therefore, false identification may occur where there is an overlapping of chromatography peaks. The improved method using LC-MS has largely overcome this drawback, by offering an additional match of MS spectrum from the standard and the corresponding compound in the sample. Furthermore, the advancement of the LC-ESI-MS/MS under MRM mode enables efficient and accurate identification of compounds in samples, by monitoring the parent and daughter ions produced by the ESI source. More importantly, due to the high sensitivity of MS detection, the LOD and LOQ of the compounds can be greatly reduced, leading to high performance in the identification of trace compounds in the samples. 

**Figure 2:**
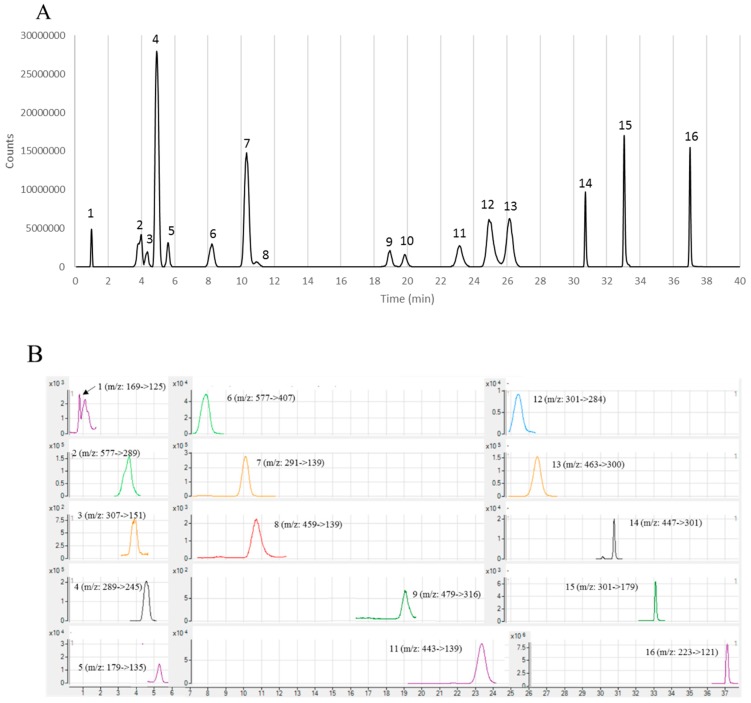
(**A**) Total ion chromatogram of the mixed standards. (**B**) Representative LC-MS chromatogram of the extracted MRM transitions of the identified phenolic compounds in feijoa peel extracts from the Unique cultivar. 1, gallic acid; 2, procyanidin B1; 3, epigallocatechin; 4, catechin; 5, caffeic acid; 6, procyanidin B2; 7, epicatechin; 8, epigallocatechin gallate; 9, myricetin-3-*O*-galactoside; 10, myricetin-3-*O*-glucoside; 11, epicatechin gallate; 12, ellagic acid; 13, quercetin-3-galactoside; 14, quercetin-3-rhamnoside; 15, quercetin; 16, flavone.

As shown in Figure 2A, 16 mixed standards were successfully separated under the optimized LC-MS program by a scan of their total ion chromatogram (TIC). Thereafter, the MRM mode of each standard was optimized using different collision energy to produce daughter ions (Table 3). A total of 15 compounds, except myricetin-3-*O*-glucoside (No. 10), were positively identified in feijoa extracts, as listed in Table 3. A representative chromatogram of the MRM transitions of the identified phenolic compounds in feijoa peel extracts was also presented in Figure 2B. For further quantification of the identified phenolics in feijoa extracts, a linear standard curve was generated for each identified compound within a serial concentration range. Good correlations of all the assessed phenolic compounds were obtained with *R*^2^ of the linearity over 0.99 (Table 3). The precision of the developed method was evaluated by inter-day and intra-day relative standard deviation (RSD%) and low values were obtained in the current study. In addition, the recovery values of all the identified compounds were between 90–110% (Table 3). All the data acquired from the method validation suggested that the optimized LC-ESI-MS/MS method was accurate and precise. It is also noted that the separation of peak 2 and 3 and peak 7 and 8 (Figure 2) were not ideal, however, it doesn’t affect the accuracy and precision of our developed method (Table 3), which indicated that the LC-ESI-MS/MS under MRM mode had a high tolerance for the interference of peak overlap, and therefore would have high performance in a wide screening of compound identification from various matrixes. 

The LOD and LOQ represent the sensitivity of the chromatography system and method. As shown in Table 3, very low LOD and LOQ were determined, indicating a high sensitivity of our method. The LOD and LOQ can vary greatly among different compounds. The lowest LOD and LOQ were observed with quercetin-3-rhamnoside (0.02 μg/L and 0.086 μg/L), while the highest was from epigallocatechin gallate (0.099 μg/mL and 0.4 μg/mL). Similar findings were from Jiao, et al. [37] and Chen, et al. [29].

#### 3.3.2. Concentrations of Phenolic Compounds in Feijoa Extracts

After the successful optimization of the LC-ESI-MS/MS method, it was applied to quantify the phenolic compounds in feijoa extracts. As shown in Table 4, a maximum of 15 phenolic compounds were quantified in feijoa flesh (13), peel (15) and whole fruit (15) extracts. A few phenolic compounds have been reported in feijoa fruits previously [17,19,54,55], while seven compounds identified in this study, namely epigallocatechin, procyanidin B2, epigallocatechin gallate, myricetin-3-*O*-galactoside, epicatechin gallate, quercetin-3-galactoside, and quercetin-3-rhamnoside, were reported in feijoa fruits for the first time. 

The concentration of each compound varied among different sample types as well as cultivars. In general, feijoa peel extracts consisted of more phenolic compounds, as well as higher compound concentrations of the identified phenolic compounds, than the flesh and whole fruit extracts. This is consistent with a former study [19] and the TPC and antioxidant activity results in Section 3.2. Among the 15 identified phenolics in feijoa peel extracts, procyanidin B1, catechin, caffeic acid and quercetin were reported in a previous study [54] focusing on feijoa waste (flesh removed) using HPLC-DAD, but the above compounds were not quantified in their research. In the current study, flavone was the most dominant phenolic compound detected in feijoa peel extracts (3302.30–5871.10 μg/g dw). Other major phenolic compounds in feijoa peel extracts, in descending order, were catechin, procyanidin B1, epicatechin, quercetin, procyanidin B2, ellagic acid and epicatechin gallate. All compounds presented in feijoa peel extracts were detected in the whole fruit extracts but at a lower concentration. Flavone (602.61–1025.49 μg/g dw), catechin (328.07–1436.49 μg/g dw) and procyanidin B1 (203.01–721.87 μg/g dw) were the dominant phenolic compounds found in feijoa whole fruit extracts. Caffeic acid and myricetin-3-*O*-galactoside were not detected in flesh extracts, and quercetin was detected in feijoa flesh (Apollo, Opal Star and Wiki Tu cultivars) at trace amount (<0.1 μg/g dw). The leading phenolic compounds found in feijoa flesh were procyanidin B1 and catechin, ranging from 305.69–776.18 and 370.48–1375.87 μg/g dw, respectively. Procyanidin B2 (19.86–64.71 μg/g dw), epicatechin (29.33–76.16 μg/g dw), ellagic acid (17.66–47.47 μg/g dw) and epicatechin gallate (11.75–25.84 μg/g dw) were also major phenolic compounds in feijoa flesh extracts. Flavone and ellagic acid were reported as important compounds in feijoa whole fruit extracts by Aoyama, Sakagami and Hatano [17], consistent with the results from the current study. However, upon closer examination of the individual flesh, peel and whole fruit extracts, it was revealed that the total feijoa flavone mostly originated from feijoa peel, with up to 1000 times higher concentrations than that in flesh. On the other hand, more than half of the total ellagic acid in feijoa fruit was found in the flesh (Table 4). Significant differences were observed among the four feijoa cultivars regarding the presence and concentration of phenolic compounds. For example, the Wiki Tu cultivar had the highest concentration of gallic acid (6.04 ± 0.06 μg/g dw) in the peel extract (similar to the Apollo cultivar, *p* > 0.05) but it comprised the lowest amount of gallic acid (1.96 ± 0.16 μg/g dw) in the flesh extract (on par with the Opal Star cultivar, *p* > 0.05). These results indicated that the phenolic profile of feijoa flesh and peel samples could be varied across different cultivars.

The total amount of phenolic compounds detected by LC-ESI-MS/MS from the extracts of four feijoa cultivars are shown in Table 4. Among the four cultivars, the phenolic content of the flesh extracts from the Opal Star and Wiki Tu cultivar (1892.86 ± 49.31 and 2368.97 ± 73.77 μg/g dw, respectively) were significantly (*p* < 0.05) higher than that of the Apollo cultivar (1283.72 ± 93.84 μg/g dw), and the Unique flesh had the lowest total concentration (859.35 ± 40.67 μg/g dw, *p* < 0.05). For the peel extracts, the Wiki Tu and Apollo cultivar was discovered to have the highest (10,461.08 ± 152.75 μg/g dw, *p* < 0.05) and lowest (7393.85 ± 237.68 μg/g dw, *p* < 0.05) total phenolic concentrations, respectively. A similar trend was observed in the whole fruit extract of the four cultivars, with the highest and lowest concentrations observed in the Wiki Tu (3743.43 ± 29.68 μg/g dw, *p* < 0.05) and Apollo (2096.72 ± 72.69 μg/g dw) cultivar, respectively. It is interesting to note that the detected total phenolic concentrations of the three extracts from the four feijoa cultivars (Table 4) were not in positive correlation with the TPC and antioxidant activity (Figure 1). For example, the Wiki Tu cultivar had a moderate TPC and antioxidant activity (Figure 1) but was found to contain the highest amount of the total phenolics detected (Table 4). This discrepancy could be due to the TPC and antioxidant activities were measured for the entire extracts, while the total concentrations of phenolics calculated from the LC-MS results were only based on the identified compounds. 

As shown in the current research, feijoa is a valuable fruit with a high content of phenolics. Its flesh would provide health benefits when consumed directly, and the peel could be a good source of phenolic-rich ingredients to be included in functional foods. Due to the relatively short season and shelf life of feijoas, there is a need to process the fruit into various food products to aid in health promotion and disease prevention year round. 

## 4. Conclusions

The current study has successfully optimized the extraction of phenolics and antioxidants from feijoa flesh, peel and whole fruit by orthogonal design, using food friendly solvent with relatively lower solvent amounts and extraction time. The TPC and antioxidant activity of feijoa extracts were determined by the FC assay and the DPPH and FRAP assays. A LC-ESI-MS/MS method was successfully developed to identify and quantify phenolic compounds in feijoa extracts.

The optimized extraction conditions for feijoa flesh and whole fruit were: extraction solvent of 70% ethanol, material to solvent ratio of 1:30, extraction temperature of 50 °C and extraction time of 30 min; while the extraction at 50 °C for 60 min using 50% ethanol and a material to solvent ratio of 1:30 was optimized for feijoa peel. Feijoa peel was found to possess higher total phenolic content and antioxidant activity than flesh and whole fruit. A total of 15 phenolic compounds were identified and quantified in feijoa extracts, and seven of them, namely epigallocatechin, procyanidin B2, epigallocatechin gallate, myricetin-3-*O*-galactoside, epicatechin gallate, quercetin-3-galactoside, and quercetin-3-rhamnoside, were reported for the first time. The Unique cultivar was discovered to have a relatively high TPC and antioxidant activity, while the Wiki Tu cultivar had the highest concentration of the detected phenolic compounds among all cultivars. Further studies are required to reveal additional phenolic compounds in feijoa. Because of the very limited study on feijoa phenolic identification, our research provides valuable information to add to the worldwide feijoa phenolic data. The observed differences among feijoa cultivars are also important to the feijoa industries regarding cultivar selection. 

## Figures and Tables

**Table 1:** Orthogonal design L9 (3^4^) and results for ethanol extraction of phenolic compounds from feijoa flesh, peel and whole fruit.

NO.	A (°C)	B	C (min)	D (Ratio)	Flesh			Peel			Whole Fruit		
					TPC (mg GAE/g dw)	Antioxidant (μM TE/g dw)		TPC (mg GAE/g dw)	Antioxidant (μM TE/g dw)		TPC (mg GAE/g dw)	Antioxidant (μM TE/g dw)	
						FRAP	DPPH		FRAP	DPPH		FRAP	DPPH
1	1 (50)	1 (30E)	1 (30)	1 (1:30)	9.66 ± 0.60	53.39 ± 2.06	56.95 ± 3.16	31.05 ± 2.29	230.8 ± 21.78	193.68 ± 4.27	12.25 ± 1.56	82.54 ± 10.10	76.14 ± 8.95
2	1	2 (50E)	2 (60)	3 (1:50)	14.58 ± 1.25	96.84 ± 7.10	86.6 ± 7.06	64.71 ± 0.05	401.63 ± 18.94	401.36 ± 1.43	22.97 ± 1.10	160.25 ± 6.25	150.96 ± 11.61
3	1	3 (70E)	3 (90)	2 (1:40)	15.08 ± 0.65	97.93 ± 2.50	90.13 ± 2.67	57.19 ± 4.17	382.00 ± 22.00	360.23 ± 18.00	24.74 ± 2.43	171.82 ± 8.38	160.56 ± 12.93
4	2 (60)	1	2	2	4.72 ± 0.03	27.22 ± 0.07	27.26 ± 0.71	30.18 ± 4.10	208.87 ± 25.28	198.90 ± 23.62	9.62 ± 1.04	62.55 ± 9.35	61.46 ± 9.69
5	2	2	3	1	11.12 ± 0.25	75.16 ± 2.57	66.74 ± 3.61	58.35 ± 0.26	386.54 ± 3.81	350.53 ± 0.64	22.84 ± 0.37	159.27 ± 0.17	148.76 ± 0.64
6	2	3	1	3	16.63 ± 1.33	107.24 ± 9.38	100.04 ± 9.51	59.39 ± 1.70	394.04 ± 9.04	371.89 ± 9.82	26.49 ± 0.75	179.53 ± 2.36	171.32 ± 2.32
7	3 (70)	1	3	3	6.12 ± 0.03	38.80 ± 0.04	43.55 ± 3.47	20.93 ± 0.55	137.12 ± 5.38	113.50 ± 1.07	9.34 ± 0.05	56.75 ± 1.34	52.16 ± 0.30
8	3	2	1	2	13.77 ± 0.65	95.47 ± 1.61	84.32 ± 2.85	64.44 ± 2.76	427.00 ± 16.23	393.32 ± 13.48	25.18 ± 2.00	178.55 ± 11.5	166.06 ± 13.86
9	3	3	2	1	18.13 ± 0.26	110.2 ± 3.00	106.27 ± 2.08	63.41 ± 0.36	407.54 ± 7.15	365.21 ± 1.61	25.82 ± 0.74	173.34 ± 0.17	161.35 ± 1.98

Factors, A—extraction temperature, B—ethanol concentration (30E, 50E and 70E—30%, 50% and 70% ethanol), C—extraction time, D—material to solvent concentration; TPC, total phenolic content; GAE, gallic acid equivalent; TE, Trolox equivalent; dw, dry weight; PRAP, ferric reducing antioxidant power; DPPH, the 2,2′-diphenyl-1-picrylhydrazyl assay.

**Table 2:** Range analysis and analysis of variance (ANOVA) of extraction factors and levels from the orthogonal experiment.

	Flesh				Peel				Whole Fruit			
Factors	A	B	C	D	A	B	C	D	A	B	C	D
*TPC*												
k1	13.11	6.83	13.36	12.97	50.99	27.39	51.63	50.94	19.99	10.40	21.31	20.31
k2	10.82	13.16	12.48	11.19	49.31	62.50	52.77	50.60	19.65	23.67	19.47	19.85
k3	12.67	16.61	10.77	12.44	49.59	60.00	45.49	48.35	20.12	25.68	18.97	19.60
Best level	A1	B3	C1	D1	A1	B2	C2	D1	A3	B3	C1	D1
R	2.28	9.78	2.58	1.78	1.68	35.11	7.28	2.60	0.47	15.28	2.34	0.70
order	BCAD				BCDA				BCDA			
*SS*	8.82	147.58	10.35	5.00	4.83	2302.28	91.93	11.95	0.35	413.45	9.08	0.77
*F* value	1.76	29.49 *	2.07	1.00	1.00	476.72 *	19.04 *	2.47	1.00	1183.80 *	26.01 *	2.20
*FRAP*												
k1	82.72	39.80	85.36	79.58	338.14	193.30	350.61	341.63	138.20	67.28	146.87	138.38
k2	69.87	89.16	78.09	73.54	330.86	405.06	340.39	340.33	133.78	166.02	132.05	137.64
k3	81.49	105.12	70.63	80.96	323.88	394.53	301.88	310.93	136.21	174.90	129.28	132.18
Best level	A1	B3	C1	D3	A1	B2	C1	D1	A1	B3	C1	D1
R	12.84	65.32	14.73	7.42	14.26	211.75	48.73	30.70	4.42	107.62	17.59	6.21
order	BCAD				BCDA				BCDA			
*SS*	301.43	6957.74	325.53	93.44	305.05	85,440.30	3961.59	1808.47	29.43	21,409.56	537.00	68.86
*F* value	3.23	74.46 *	3.48	1.00	1.00	280.08 *	12.99	5.93	1.00	727.53 *	18.25	2.34
*DPPH*												
k1	77.89	42.59	80.44	76.65	318.42	168.69	319.63	303.14	129.22	63.25	137.84	128.75
k2	64.68	79.22	73.38	67.24	307.11	381.74	321.82	317.48	127.18	155.26	124.59	129.36
k3	78.05	98.81	66.81	76.73	290.68	365.78	274.75	295.58	126.52	164.41	120.49	124.82
Best level	A3	B3	C1	D3	A1	B2	C2	D2	A1	B3	C1	D2
R	13.37	56.23	13.63	9.49	27.75	213.04	47.07	21.90	2.70	101.15	17.35	4.54
order	BCAD				BCAD				BCDA			
*SS*	353.43	4887.39	278.62	178.77	1167.79	84,484.49	4234.39	742.40	11.88	18,780.65	493.24	36.47
*F* value	1.98	27.34 *	1.56	1.00	1.57	113.80 *	5.70	1.00	1.00	1581.48 *	41.53 *	3.07

Factors, A—extraction temperature, B—ethanol concentration, C—extraction time, D—material to solvent concentration; TPC, total phenolic content; FRAP, ferric reducing antioxidant power; DPPH, the 2,2′-diphenyl-1-picrylhydrazyl assay; Range analysis, k-averaged value of each level of the factors (k1, k2, k3 for level 1, 2, 3, respectively), the biggest k value indicates the best level for each factor; R, range between the maximum and minimum k value, a larger R value suggests a more important role of the factor in the extraction process; order, influential order of the factors, determined by the descending order of R value; *SS*, sum of squares; *F* value, obtained from ANOVA Fisher’s F-test, F (2,2) 95% = 19; *, *p* < 0.05.

**Table 3:** Accuracy and precision of the qualification and quantification method of phenolic compounds.

		Parent Ion (*m*/*z*)	Ion Mode	Daughter Ion (Energy)		Regression Equation	*R* ^2^	Linear Range (μg/mL)	LOD (μg/mL)	LOQ (μg/mL)	Precision (RSD%)		Recovery
				(1)	(2)						Inter Day	Intra Day	
1	Gallic acid	169	neg	125 (10 V)	79 (25 V)	y = 129.3914x − 2.3946	0.9998	0.055–2.198	0.003434	0.013736	0.76	3.82	1.08
2	Procyanidin B1	577	neg	289 (15 V)	245 (15 V)	y = 171.4361x − 43.0696	0.9998	0.165–26.376	0.002576	0.010302	1.51	3.05	1.02
3	Epigallocatechin	307	pos	139 (3 V)	151 (11 V)	y = 286.2960x − 13.4304	0.9993	0.070–2.812	0.008791	0.035165	2.11	2.35	1.04
4	Catechin	289	neg	245 (10 V)	203 (15 V)	y = 318.1257x + 247.7167	0.9963	0.550–65.934	0.002146	0.008585	4.25	0.42	0.94
5	Caffeic acid	179	neg	135 (10 V)	89 (40 V)	y = 261.8032x + 8.4716	0.9976	0.275–3.294	0.006868	0.027473	5.30	0.03	0.96
6	Procyanidin B2	577	neg	289 (15 V)	407 (15 V)	y = 174.7724x − 41.7501	0.9995	0.110–17.584	0.001717	0.006868	1.88	0.51	1.04
7	Epicatechin	291	pos	139 (13 V)	123 (30 V)	y = 348.7135x + 124.5464	0.9982	0.296–47.296	0.002309	0.009238	1.79	6.40	0.99
8	Epigallocatechin gallate	459	pos	139 (2 V)	289 (23 V)	y = 125.6769x − 35.9066	0.9995	0.397–7.930	0.099073	0.396291	2.60	0.89	1.11
9	Myricetin-3-*O*-galactoside	479	neg	316 (20 V)	271 (40 V)	y = 242.9794x − 20.7572	0.9999	0.110–10.990	0.000859	0.003434	3.10	1.66	1.02
10	Myricetin-3-*O*-glucoside	479	neg	316 (20 V)	271 (40 V)	-	-	-	-	-	-	-	-
11	Epicatechin gallate	443	pos	139 (20 V)	123 (14 V)	y = 165.9345x − 27.1226	0.9993	0.101–8.088	0.017857	0.071429	1.51	7.09	0.94
12	Ellagic acid	301	neg	301 (0 V)	284 (25 V)	y = 153.4873x + 4.6456	0.9978	0.571–11.429	0.003159	0.012637	0.24	8.73	1.06
13	Quercetin-3-galactoside	463	neg	300 (18 V)	270 (40 V)	y = 179.8942x − 3.5259	0.9997	0.275–21.980	0.008585	0.034341	1.58	9.15	0.98
14	Quercetin-3-rhamnoside	447	neg	301 (10 V)	300 (25 V)	y = 248.6660x + 7.2576	0.9989	0.055–10.99	2.15 × 10^−5^	8.59 × 10^−5^	2.81	7.90	0.98
15	Quercetin	301	neg	151 (20 V)	179 (10 V)	y = 738.5890x + 27.1359	0.9969	0.055–4.396	0.000215	0.000859	0.71	3.91	0.94
16	Flavone	223	pos	121 (17 V)	77 (33 V)	y = 85.1514x + 14.9630	0.9968	0.275–43.96	0.000107	0.000429	1.18	0.58	0.98

LOD, limit of detection; LOQ, limit of quantification; RSD%, relative standard deviation; Ion mode, neg-negative, pos-positive.

**Table 4:** Concentrations of phenolic compounds in feijoa flesh, peel and whole fruit of four cultivars.

	Flesh (μg/g dw)				Peel (μg/g dw)				Whole Fruit (μg/g dw)			
Compounds	A	U	OS	WT	A	U	OS	WT	A	U	OS	WT
Gallic acid	4.31 ± 0.27 ^ef^	3.8 ± 0.45 ^de^	2.42 ± 0.02 ^bc^	1.96 ± 0.16 ^ab^	5.54 ± 0.1 ^gh^	5.03 ± 0.09 ^fg^	3.92 ± 0.06 ^de^	6.04 ± 0.06 ^h^	2.56 ± 0.18 ^bc^	1.21 ± 0.01 ^a^	3.18 ± 0.12 ^cd^	2.67 ± 0.08 ^bc^
Procyanidin B1	473.29 ± 23.13 ^cd^	305.69 ± 10.22 ^ab^	488.87 ± 8.98 ^cd^	776.18 ± 36.04 ^e^	1195.15 ± 96.17 ^f^	565.16 ± 21.78 ^d^	1070.88 ± 21.23 ^f^	833.34 ± 4.78 ^e^	371.27 ± 19.59 ^bc^	203.01 ± 9.34 ^a^	551.58 ± 7.45 ^d^	721.87 ± 18.7 ^e^
Epigallocatechin	3.73 ± 0.2 ^a^	4.3 ± 0.34 ^b^	6.94 ± 0.12 ^ab^	6.16 ± 0.35 ^ab^	9.16 ± 0.33 ^b^	20.97 ± 0.38 ^d^	39.26 ± 2.46 ^e^	13.49 ± 0.05 ^c^	6.18 ± 0.37 ^ab^	6.96 ± 1.06 ^ab^	9.8 ± 0.45 ^bc^	10.48 ± 0.2 ^bc^
Catechin	651.31 ± 63.17 ^b^	370.48 ± 20.89 ^ab^	1191.82 ± 36.27 ^c^	1375.87 ± 30.81 ^cde^	1311.2 ± 88.58 ^cd^	698.29 ± 19.17 ^b^	1829.91 ± 39.67 ^f^	1551.65 ± 11.11 ^e^	608.97 ± 28.09 ^b^	328.07 ± 15.6 ^a^	1436.49 ± 31.06 ^de^	1428.05 ± 12.5 ^de^
Caffeic acid	nd	nd	nd	nd	16.15 ± 0.56 ^c^	43.82 ± 4.02 ^e^	19.1 ± 0.89 ^c^	34.65 ± 0.2 ^d^	4.4 ± 1 ^ab^	9.05 ± 0.75 ^b^	4.36 ± 0.63 ^ab^	6.56 ± 0.77 ^ab^
Procyanidin B2	42.98 ± 4.06 ^ab^	19.86 ± 1.04 ^a^	57.73 ± 0.46 ^b^	64.71 ± 3.4 ^b^	338.62 ± 15.1 ^f^	230.43 ± 5.5 ^d^	317.07 ± 3.03 ^ef^	312.95 ± 2.46 ^e^	64.72 ± 3.58 ^b^	42.01 ± 2.44 ^ab^	90.83 ± 3.33 ^c^	106.04 ± 0.21 ^c^
Epicatechin	29.33 ± 4.21 ^a^	76.16 ± 2.56 ^b^	73.42 ± 0.46 ^b^	73.42 ± 0.46 ^b^	460.32 ± 20.25 ^d^	691.63 ± 11.42 ^f^	570.24 ± 0.77 ^e^	570.24 ± 0.77 ^e^	84.78 ± 5.54 ^b^	167.23 ± 3.94 ^c^	176.8 ± 0.63 ^c^	176.8 ± 0.63 ^c^
Epigallocatechin gallate	8.74 ± 0.17 ^a^	10.47 ± 0.06 ^ab^	12.26 ± 0.04 ^b^	10.41 ± 0.14 ^ab^	13.61 ± 0.15 ^b^	24.82 ± 0.9 ^d^	17.44 ± 0.93 ^c^	13.17 ± 0.62 ^b^	10.63 ± 0.1 ^ab^	11.54 ± 1.31 ^ab^	11.62 ± 0.46 ^ab^	11.3 ± 0.49 ^ab^
Myricetin-3-*O*-galactoside	nd	nd	nd	nd	nd	5.87 ± 0.17 ^c^	4.04 ± 0.09 ^b^	3.84 ± 0.27 ^b^	nd	3.2 ± 0.02 ^a^	nd	2.81 ± 0.25 ^ab^
Ellagic acid	32.54 ± 0.59 ^ab^	47.47 ± 5.47 ^bc^	21.58 ± 1.02 ^a^	17.66 ± 2.16 ^a^	164.86 ± 5.46 ^f^	211.68 ± 3.84 ^g^	120.76 ± 5.14 ^e^	156 ± 4.08 ^f^	61.32 ± 4.9 ^cd^	72.64 ± 3.87 ^d^	50.3 ± 3.89 ^bc^	35.09 ± 0.52 ^ab^
Epicatechin gallate	23.3 ± 0.59 ^b^	11.75 ± 0.6 ^a^	25.84 ± 1.58 ^b^	20.13 ± 1.06 ^b^	165.29 ± 1.72 ^f^	93.56 ± 1.42 ^d^	116.96 ± 3.62 ^e^	110.67 ± 0.21 ^e^	40.57 ± 1.62 ^c^	17.92 ± 0.33 ^ab^	38.12 ± 1.83 ^c^	39.54 ± 2.19 ^c^
Quercetin-3-galactoside	5.03 ± 0.33 ^a^	2.44 ± 0.18 ^a^	4.34 ± 0.26 ^a^	6.34 ± 0.41 ^a^	339.49 ± 8.13 ^e^	492.9 ± 4.21 ^g^	429.71 ± 1.51 ^f^	816.82 ± 7.54 ^h^	64.98 ± 1.49 ^b^	106.97 ± 7.83 ^c^	61.64 ± 4.9 ^b^	138.96 ± 0.22 ^d^
Quercetin-3-rhamnoside	5.08 ± 0.21 ^a^	5.33 ± 0.24 ^a^	7.11 ± 0.03 ^a^	11.66 ± 0.3 ^bc^	41.54 ± 1.24 ^f^	88.11 ± 0.87 ^h^	58.45 ± 0.66 ^g^	98.89 ± 0.61 ^i^	10.7 ± 0.18 ^b^	15.82 ± 0.76 ^d^	14.41 ± 1.37 ^cd^	23.84 ± 0.96 ^e^
Quercetin	<0.1	<0.1	<0.1	<0.1	30.61 ± 0.81 ^c^	62.74 ± 1.86 ^d^	34.23 ± 0.49 ^c^	93.05 ± 3.64 ^e^	3.97 ± 0.03 ^a^	6.28 ± 0.23 ^a^	4.04 ± 0.39 ^a^	13.93 ± 0.46 ^b^
Flavone	4.07 ± 0.1 ^a^	1.6 ± 0.17 ^a^	0.52 ± 0.12 ^a^	4.48 ± 0.21 ^a^	3302.3 ± 2.68 ^d^	5871.1 ± 85.25 ^e^	3435.21 ± 6.71 ^d^	5846.27 ± 142.3 ^e^	761.69 ± 16.78 ^b^	1208.06 ± 13.42 ^c^	602.61 ± 17.45 ^b^	1025.49 ± 21.48 ^c^
Total	1283.72 ± 93.84 ^b^	859.35 ± 40.67 ^a^	1892.86 ± 49.31 ^c^	2368.97 ± 73.77 ^c^	7393.85 ± 237.68 ^f^	9106.11 ± 150.02 ^g^	8067.18 ± 85.94 ^h^	10,461.08 ± 152.75 ^i^	2096.72 ± 72.69 ^c^	2199.98 ± 51.06 ^c^	3055.77 ± 16.33 ^d^	3743.43 ± 29.68 ^e^

A, Apollo cultivar; U, Unique cultivar, OS, Opal Star cultivar; WT, Wiki Tu cultivar; dw, dry weight; ^abcdefghi^, indicators for significance among samples and cultivars (*p* < 0.05).
